# Supplementary material for: Mirror-like Bright Al-Mn Coatings Electrodeposition from 1-Ethyl-3 Methylimidazolium Chloride-AlCl3-MnCl2 Ionic Liquids with Pyridine Derivatives
Source: Materials (Basel). 2021 Oct 19;14(20):6226. doi: 10.3390/ma14206226 (PMC8539174; doi:10.3390/ma14206226)
Supplement: Supplementary file 1 [file materials-14-06226-s001.zip › materials-1400404-supplementary.pdf]

Supporting Information

Mirror-like Bright Al-Mn Coatings Electrodeposition from 1-Ethyl-3 Methylimidazolium Chloride-$AlCl_3$-$MnCl_2$ Ionic Liquids with Pyridine Derivatives

Dong Peng [1], Da Long Cong [1], Kai Qiang Song [1], Xing Xing Ding [1], Xuan Wang [1], Yi Xin Bai [1], Xin Rui Yang [1], Chang Qing Yin [2], Yu Xin Zhang [2], Jin Song Rao [2], Min Zhang [1,*] and Zhong Sheng Li [1,*]

[1] Southwest Institute of Technology and Engineering, Chongqing 400039, P.R China; pd2019@126.com (D.P.); congdl09@163.com (D.C); scut_song@163.com (K.S.); sc126789@gmail.com (X.D.); xuan123_wang@sina.com (X.W.); bitbyx@163.com (Y.B); yxr67938543@163.com (X.Y.)

[2] College of Material Science and Engineering, Chongqing University, Chongqing 400044, P.R China; 2972513971@qq.com (C.Y.); zhangyuxin@cqu.edu.cn (Y.Z.); rjs@cqu.edu.cn (J.R.)

* Correspondence: (M.Z.); zhongshli@163.com (Z.L.); Tel.: +86-23-68792314

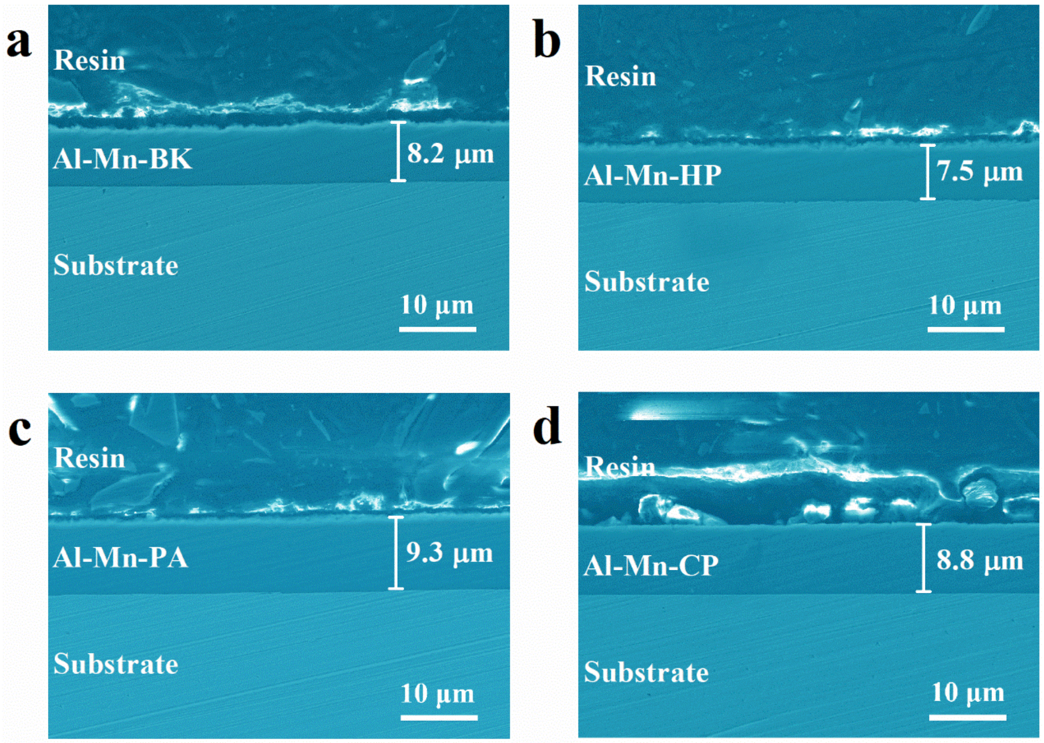

Figure S1. Cross-section morphology of the (a) Al-Mn-BK, (b) Al-Mn-HP, (c) Al-Mn-PA, and (d) Al-Mn-CP coatings.

The preferred orientation of various Al-Mn coatings was determined at the quantitative level by the determination of "Texture Coefficient" (*TC (hkl)*) and "Relative Texture Coefficient" (*RTC (hkl)*) using the reagent grade fine aluminum powder as the reference sample [1,2].

$$R_1(hkl) = \frac{I(hkl)_s}{\sum I(hkl)_s} \times 100 \quad (1)$$

where *I(hkl)s* is the intensity of the *(hkl)* reflection, and *ΣI(hkl)s* is the sum of all intensities of all the reflections monitored. *S* refers to the investigated sample.

$$R_2(hkl) = \frac{I(hkl)_p}{\sum I(hkl)_p} \times 100 \quad (2)$$

The subscript *P* represents the reference sample.

$$TC(hkl) = \frac{R_1(hkl)}{R_2(hkl)} \quad (3)$$

A value of *TC (hkl)* greater than 1 indicates a preferred orientation of the *(hkl)* reflection compared with the random distribution of grains in the reference sample.

$$RTC(hkl) = \frac{TC(hkl)}{\sum TC(hkl)} \quad (4)$$

The *RTC(hkl)* coefficient expresses the intensity of a given orientation *(hkl)* relative to reference

sample, as a percentage of the intensity of all orientations parallel to the surface of the investigated sample.

Table S1. Texture calculations for various Al-Mn coatings obtained from the XRD patterns in Fig. 4.

		**Plane *(hkl)***				**ΣI**
		**(111)**	**(200)**	**(220)**	**(311)**	
Intensity	Al	1247850	1289499	556051	567645	3661045
	Al-Mn-BK	973	4648	3512	207	9340
	Al-Mn-HP	1335	14546	1276	105	17261
	Al-Mn-PA	761	94784	0	0	95545
	Al-Mn-CP	656	105962	0	0	106618
R_1%	Al-Mn-BK	10.4164	49.7660	37.5982	2.2194	
	Al-Mn-HP	7.7326	84.2693	7.3922	0.6059	
	Al-Mn-PA	0.7965	99.2034	0.0	0.0	
	Al-Mn-CP	0.62	99.38	0.0	0.0	
R_2%	Al	34.0845	35.2221	15.183	15.5050	
TC(hkl)	Al-Mn-BK	0.3056	1.4129	2.4754	0.1431	
	Al-Mn-HP	0.2269	2.3925	0.4867	0.0391	
	Al-Mn-PA	0.0234	2.8165	0.0	0.0	
	Al-Mn-CP	0.0180	2.8217	0.0	0.0	
RTC(hkl) *(%)*	Al-Mn-BK	7.0463	32.5772	57.0762	3.3004	
	Al-Mn-HP	7.2132	76.0696	15.4747	1.2425	
	Al-Mn-PA	0.8229	99.1771	0.0	0.0	
	Al-Mn-CP	0.6354	99.3646	0.0	0.0	

Table S2. Average roughness of Al-Mn coatings.

**Materials**	Al-Mn-BK	Al-Mn-HP	Al-Mn-PA	Al-Mn-CP
**Average roughness (nm)**	243	239	76	18

References

1. Berube, L.P.; Esperance, G.L. A quantitative method of determining of the degree of texture of zinc electrodeposits. Journal of The Electrochemical Society 1989, 136, 2314–2315.
2. Yang, J.; Chang, L.; Jiang, L.; Wang, K.; Huang, L.; He, Z.; Shao, H.; Wang, J.; Cao, C.N. Electrodeposition of Al-Mn-Zr ternary alloy films from the Lewis acidic aluminum chloride-1-ethyl-3-methylimidazolium chloride ionic liquid and their corrosion properties. *Surface and Coatings Technology* **2017**, *321*, 45-51.
